# Supplementary material for: TA-RNN: an attention-based time-aware recurrent neural network architecture for electronic health records
Source: Bioinformatics. 2024 Jun 28;40(Suppl 1):i169–79. doi: 10.1093/bioinformatics/btae264 (PMC11211851; doi:10.1093/bioinformatics/btae264)
Supplement: btae264_Supplementary_Data [file btae264_supplementary_data.zip › btae264_Supplementary_Data/Bozdag.207.sup.1.pdf]

Supplemental Table 1 List of ADNI data features used to train the models in the first experimental setup where models were trained on 70% of ADNI data and tested on the 30% held-out ADNI data. MRI: Magnetic Resonance Imaging.

**Feature name**	**Description**	**Category**	**Type**
**CDRSB**	Clinical Dementia Rating Scale–Sum of Boxes	Cognitive performance	Longitudinal
**ADAS11**	Alzheimer’s Disease Assessment Scale 11	Cognitive performance	Longitudinal
**ADAS13**	Alzheimer’s Disease Assessment Scale 13	Cognitive performance	Longitudinal
**ADASQ4**	Alzheimer’s Disease Assessment Scale Q4	Cognitive performance	Longitudinal
**MMSE**	Mini-Mental State Examination	Cognitive performance	Longitudinal
**RAVLT.immediate**	Rey Auditory Verbal Learning Test (Immediate recall)	Cognitive performance	Longitudinal
**RAVLT.learning**	Rey Auditory Verbal Learning Test (Learning)	Cognitive performance	Longitudinal
**RAVLT.forgetting**	Rey Auditory Verbal Learning Test (Forgetting)	Cognitive performance	Longitudinal
**RAVLT.perc.forgetting**	Rey Auditory Verbal Learning Test (Percent Forgetting)	Cognitive performance	Longitudinal
**LDELTOTAL**	The logical memory delayed recall total	Cognitive performance	Longitudinal
**TRABSCOR**	Trail Making Test-B	Cognitive performance	Longitudinal
**FAQ**	Functional Activities Questionnaire	Cognitive performance	Longitudinal
**Ventricles**	Ventricles measurement	MRI	Longitudinal
**Hippocampus**	Hippocampus measurement	MRI	Longitudinal
**WholeBrain**	Whole Brain measurement	MRI	Longitudinal
**Entorhinal**	Entorhinal cortex measurement	MRI	Longitudinal
**Fusiform**	Fusiform Gyrus	MRI	Longitudinal
**MidTemp**	Scan of the middle temporal artery	MRI	Longitudinal
**ICV**	Intracranial volume	MRI	Longitudinal
**AGE***	Age of patient	Demographic	Longitudinal
**PTGENDER**	Gender of patient	Demographic	Cross-sectional
**PTEDUCAT**	Education years of patient	Demographic	Cross-sectional
**PTETHCAT**	Ethnicity of patient	Demographic	Cross-sectional
**PTRACCAT**	Race of patient	Demographic	Cross-sectional
**APOE4**	Number of ε4 allele	Demographic	Cross-sectional

*AGE was used only with RF, SVM, and PPAD.

Supplemental Table 2 Statistics of ADNI data for each scenario in all splits in the first experimental setup. For each scenario, $x \rightarrow y$ means that the model was trained using x visits and tested the diagnosis at the next y^{th} visit ahead.

Split	Scenario	Train Data			Test Data		
		# Samples	# MCI	# AD	# Samples	# MCI	# AD
1	**2→1**	774	499	275	343	211	132
	**2→2**	672	378	294	267	159	108
	**2→3**	522	291	231	185	124	61
	**2→4**	359	219	140	137	84	53
	**3→1**	774	438	336	267	159	108
	**3→2**	672	349	323	185	124	61
	**3→3**	522	261	261	137	84	53
	**3→4**	359	203	156	87	47	40
	**5→1**	774	379	395	137	84	53
	**5→2**	672	303	369	87	47	40
	**5→3**	522	239	283	55	32	23
	**5→4**	359	195	164	41	23	18
	**6→1**	774	363	411	87	47	40
	**6→2**	672	297	375	55	32	23
	**6→3**	522	237	285	41	23	18
	**6→4**	359	193	166	26	17	9
2	**2→1**	780	499	281	338	212	126
	**2→2**	677	380	297	263	157	106
	**2→3**	526	294	232	181	121	60
	**2→4**	363	223	140	133	80	53
	**3→1**	780	441	339	263	157	106
	**3→2**	677	352	325	181	121	60
	**3→3**	526	265	261	133	80	53
	**3→4**	363	202	161	83	48	35
	**5→1**	780	384	396	133	80	53
	**5→2**	677	302	375	83	48	35
	**5→3**	526	238	288	52	33	19
	**5→4**	363	193	170	39	25	14
	**6→1**	780	363	417	83	48	35
	**6→2**	677	296	381	52	33	19
	**6→3**	526	235	291	39	25	14
	**6→4**	363	192	171	24	18	6
3	**2→1**	783	483	300	335	228	107
	**2→2**	680	373	307	260	164	96
	**2→3**	529	286	243	179	129	50
	**2→4**	365	215	150	131	88	43
	**3→1**	783	434	349	260	164	96
	**3→2**	680	344	336	179	129	50
	**3→3**	529	257	272	131	88	43
	**3→4**	365	200	165	81	50	31
	**5→1**	783	376	407	131	88	4343
	**5→2**	680	300	380	81	50	31
	**5→3**	529	240	289	50	31	19
	**5→4**	365	195	170	37	23	14
	**6→1**	783	361	422	81	50	31
	**6→2**	680	298	382	50	31	19
	**6→3**	529	237	292	37	23	14
	**6→4**	365	194	171	22	16	6

Supplemental Table 3 List of NACC data features for the second experimental setup, where models were trained on ADNI data and tested on NACC data.

Feature name	Corresponding feature in ADNI
**CDRSUM**	CDRSB
**NACCMMSE**	MMSE
**MEMUNITS**	LDELTOTAL
**FAQ***	FAQ
**NACCAGE**	AGE
**SEX**	PTGENDER
**EDUC**	PTEDUCAT
**HISPANIC**	PTETHCAT
**RACE**	PTRACCAT
**NACCNE4S**	APOE4

* NACC dataset does not have FAQ feature, but it has the raw data that can be used to get it based on the following equation:

FAQ = BILLS+ TAXES+ SHOPPING+ GAMES+STOVE+MEALPREP+EVENTS+PAYATTN+ REMDATES+TRAVEL

The value of each feature that contributes FAQ value has the following range (0 = Normal, 1 = Has difficulty but does by self, 2 = Requires assistance, 3 = Dependent).

Supplemental Table 4 Statistics of ADNI as train data and NACC as test data for each scenario in the second experimental setup. For each scenario, $x \rightarrow y$ means that the model was trained using x visits and tested the diagnosis at the next y^{th} visit ahead.

Scenario	Train Data (ADNI)			Test Data (NACC)		
	**# Samples**	**# MCI**	**# AD**	**# Samples**	**# MCI**	**# AD**
**2→1**	1205	745	460	6118	1447	4671
**2→2**	1040	569	471	4399	869	3530
**2→3**	821	439	382	3049	522	2527
**2→4**	568	335	233	2117	317	1800
**3→1**	1205	666	539	4399	869	3530
**3→2**	1040	516	524	3049	522	2527
**3→3**	821	399	422	2117	317	1800
**3→4**	568	307	261	1414	19	1216
**5→1**	1205	573	632	2117	317	1800
**5→2**	1040	448	592	1414	198	1216
**5→3**	821	361	460	889	121	768
**5→4**	568	292	276	551	71	480
**6→1**	1205	545	660	1414	198	1216
**6→2**	1040	438	602	889	121	768

**6→3**	821	356	465	551	71	480
**6→4**	568	288	280	306	44	262

Supplemental Table 5 Optimal hyperparameter values of TA-RNN for each scenario in all splits in the first experimental setup. For each scenario, x→1 means that the model was trained using x visits and tested the diagnosis at the next visit.

**Data split**	**Scenario**	**RNN cell**	**Batch size**	**Epochs**	**Dropout rate**	**L2**	**Hidden size**	**Embedding size**
**1**	2→1	Bi-GRU	32	50	0.2	1E-7	16	19
	3→1	Bi-GRU	32	50	0.2	1E-7	16	19
	5→1	Bi-GRU	32	50	0.2	1E-7	16	19
	6→1	Bi-LSTM	32	50	0.2	1E-7	16	19
**2**	2→1	Bi-LSTM	32	50	0.2	1E-7	16	19
	3→1	Bi-GRU	32	40	0.2	1E-7	16	19
	5→1	Bi-GRU	32	50	0.2	1E-7	16	19
	6→1	Bi-LSTM	32	40	0.2	1E-7	16	19
**3**	2→1	Bi-GRU	32	30	0.2	1E-7	16	19
	3→1	Bi-GRU	32	50	0.2	1E-7	16	19
	5→1	Bi-GRU	32	50	0.2	1E-7	16	19
	6→1	Bi-LSTM	32	40	0.2	1E-7	16	19

Supplemental Table 6 Optimal hyperparameter values of TA-RNN for each scenario in the second experimental setup. For each scenario, x→1 means that the model was trained using x visits and tested the diagnosis at the next visit.

**Scenario**	**RNN cell**	**Batch size**	**Epochs**	**Dropout rate**	**L2**	**Hidden Size**	**Embedding size**
**2→1**	Bi-GRU	32	50	0.2	1E-7	16	4
**3→1**	Bi-LSTM	32	50	0.2	1E-7	16	4
**5→1**	Bi-GRU	32	50	0.2	1E-7	16	4
**6→1**	Bi-LSTM	32	50	0.2	1E-7	16	4

Supplemental Table 7 Optimal hyperparameter values of TA-RNN-AE for each scenario in all splits in the first experimental setup. For each scenario, $x \rightarrow y$ means that the model was trained using x visits and tested the diagnosis at the next y^{th} visit ahead.

Data split	Scenario	RNN cell	Batch size	Epochs	Dropout rate	L2	Hidden size	Embedding size
**1**	2→2	Bi-LSTM	4	50	0.0	1E-3	16	19
	2→3	Bi-LSTM	4	50	0.2	1E-7	16	19
	2→4	Bi-GRU	16	50	0.2	1E-3	16	19
	3→2	Bi-GRU	8	30	0.3	1E-7	16	19
	3→3	Bi-GRU	4	30	0.2	1E-7	16	19
	3→4	Bi-GRU	8	40	0.4	1E-7	16	19
	5→2	Bi-GRU	2	50	0.1	1E-7	16	19
	5→3	Bi-GRU	32	50	0.2	1E-7	16	19
	5→4	Bi-GRU	32	50	0.2	1E-7	16	19
	6→2	Bi-GRU	32	50	0.2	1E-7	16	19
	6→3	Bi-LSTM	2	50	0.3	1E-7	16	19
	6→4	Bi-GRU	32	50	0.2	1E-7	16	19
**2**	2→2	Bi-GRU	32	20	0.2	1E-7	16	19
	2→3	Bi-GRU	32	30	0.2	1E-7	16	19
	2→4	Bi-GRU	32	20	0.2	1E-7	16	19
	3→2	Bi-GRU	32	20	0.2	1E-5	16	19
	3→3	GRU	8	40	0.0	1E-7	16	19
	3→4	Bi-GRU	64	30	0.0	1E-5	16	19
	5→2	Bi-LSTM	16	100	0.1	1E-5	16	19
	5→3	Bi-GRU	32	20	0.2	1E-7	16	19
	5→4	GRU	32	50	0.2	1E-7	16	19
	6→2	Bi-GRU	32	100	0.2	1E-7	16	19
	6→3	Bi-GRU	8	30	0.3	1E-5	16	19
	6→4	Bi-GRU	32	40	0.2	1E-7	16	19
**3**	**2→2**	**Bi-GRU**	**32**	**50**	**0.2**	**1E-7**	**16**	**19**
	2→3	Bi-GRU	32	50	0.2	1E-7	16	19

	**2→4**	**Bi-GRU**	**32**	**50**	**0.2**	**1E-7**	**16**	**19**
	3→2	Bi-GRU	64	50	0.0	1E-3	16	19
	**3→3**	**Bi-GRU**	**8**	**30**	**0.1**	**1E-7**	**16**	**19**
	3→4	Bi-GRU	4	50	0.0	1E-5	16	19
	**5→2**	**Bi-GRU**	**2**	**40**	**0.3**	**1E-5**	**16**	**19**
	5→3	Bi-GRU	32	50	0.2	1E-7	16	19
	**5→4**	**Bi-GRU**	**32**	**50**	**0.2**	**1E-7**	**16**	**19**
	6→2	Bi-GRU	32	50	0.2	1E-7	16	19
	**6→3**	**Bi-GRU**	**16**	**100**	**0.3**	**1E-5**	**16**	**19**
	6→4	Bi-GRU	32	50	0.2	1E-7	16	19

Supplemental Table 8 Optimal hyperparameter values of TA-RNN-AE for each scenario in the second experimental setup. For each scenario, $x \rightarrow y$ means that the model was trained using x visits and tested the diagnosis at the next y^{th} visit ahead.

Scenario	RNN cell	Batch size	Epochs	Dropout rate	L2	Hidden size	Embedding size
**2→2**	Bi-LSTM	32	20	0.0	1E-7	16	4
**2→3**	Bi-LSTM	32	10	0.0	1E-7	16	4
**2→4**	Bi-GRU	32	10	0.0	1E-7	16	4
**3→2**	Bi-GRU	64	20	0.2	1E-7	32	4
**3→3**	Bi-GRU	32	30	0.2	1E-7	16	4
**3→4**	Bi-GRU	32	40	0.0	1E-7	16	4
**5→2**	GRU	2	10	0.0	1E-3	32	4
**5→3**	Bi-GRU	32	30	0.3	1E-7	16	4
**5→4**	Bi-GRU	2	10	0.0	1E-7	16	4
**6→2**	Bi-LSTM	32	100	0.0	1E-7	16	4
**6→3**	Bi-GRU	32	10	0.2	1E-7	16	4
**6→4**	Bi-GRU	32	50	0.2	1E-7	16	4

Supplemental Table 9 Statistics of MIMIC-III data in the third experimental setup.

Train Data			Test Data		
**# Samples**	**# Negative**	**# Positive**	**# Samples**	**# Negative**	**# Positive**
4747	2968	1779	2262	1414	848

Supplemental Table 10 Optimal hyperparameter values of TA-RNN in the third experimental setup.

RNN cell	Batch size	Epochs	Dropout rate	L2	Hidden size	Embedding size
**GRU**	8	5	0.5	1E-7	128	128

Supplemental Table 11 Results of t-test comparing the F2 scores of TA-RNN against other models for the first experimental setup (see Figure 3A). *P*-values indicate the statistical significance of differences in performance. Significant p-values (i.e., ≤0.05) are shown in bold.

Scenario	RF vs. TA-RNN	SVM vs. TA-RNN	T-LSTM vs. TA-RNN	PPAD vs. TA-RNN
**2→1**	**8.3 E-8**	**4.5 E-7**	**1.04 E-9**	0.210
**3→1**	**2.8 E-25**	**1.6 E-7**	**8.5 E-6**	**0.011**
**5→1**	**3.8 E-41**	**2.0 E-27**	**4.1 E-10**	0.063
**6→1**	**3.3 E-22**	**1.7 E-16**	**6.0 E-4**	0.6

Supplemental Table 12 Results of t-test comparing the F2 scores of TA-RNN against other models for the second experimental setup (see Figure 3B). *P*-values indicate the statistical significance of differences in performance. Significant p-values (i.e., ≤0.05) are shown in bold.

Scenario	RF vs. TA-RNN	SVM vs. TA-RNN	T-LSTM vs. TA-RNN	PPAD vs. TA-RNN
**2→1**	**3.6 E-40**	**1.9 E-35**	**1.2 E-30**	**3.0 E-4**
**3→1**	**5.2 E-47**	**1.5 E-44**	**1.2 E-21**	0.31
**5→1**	**2.7 E-51**	**2.8 E-54**	0.36	0.41
**6→1**	**1.3 E-62**	**1.5 E-65**	**1.5 E-7**	1.0

Supplemental Table 13 Results of t-test comparing the F2 scores of TA-RNN-AE against other models for the first experimental setup (see Figure 4A-D). *P*-values indicate the statistical significance of differences in performance. Significant p-values (i.e., ≤0.05) are shown in bold.

Scenario	RF vs TA-RNN-AE	SVM vs TA-RNN-AE	PPAD-AE vs TA-RNN-AE
**2→2**	**2.0 E-22**	**4.3 E-5**	**0.018**
**2→3**	**4.0 E-18**	**8.0 E-6**	0.21
**2→4**	**4.1 E-22**	**2.2 E-21**	**0.006**
**3→2**	**4.1 E-19**	**1.2 E-10**	0.26

**3→3**	**9.0 E-21**	**2.4 E-10**	0.12
**3→4**	**1.7 E-6**	**5.4 E-12**	0.6
**5→2**	**1.2 E-13**	**5.1 E-17**	**0.021**
**5→3**	0.3	0.63	1.0
**5→4**	0.09	**6.6 E-6**	0.35
**6→2**	**1.0 E-4**	**1.9 E-6**	0.57
**6→3**	**7.0 E-7**	**5.5 E-5**	0.14
**6→4**	0.28	**0.006**	0.58

Supplemental Table 14 Results of t-tests comparing the F2 scores of TA-RNN-AE against other models for the second experimental setup (see Figure 4E-H). *P*-values indicate the statistical significance of differences in performance. Significant p-values (i.e., ≤0.05) are shown in bold.

**Scenario**	**RF vs TA-RNN-AE**	**SVM vs TA-RNN-AE**	**PPAD-AE vs TA-RNN-AE**
**2→2**	**7.5 E-35**	**5.6 E-33**	**0.016**
**2→3**	**3.2 E-30**	**2.1 E-34**	0.11
**2→4**	**2.1 E-8**	**2.9 E-10**	1.0
**3→2**	**1.6 E-26**	**9.5 E-34**	**0.034**
**3→3**	**1.0 E-3**	**1.4 E-25**	**2.4 E-5**
**3→4**	**3.1 E-27**	**3.3 E-35**	**1.9 E-9**
**5→2**	**5.3 E-34**	**7.7 E-45**	0.084
**5→3**	**1.5 E-31**	**1.6 E-40**	0.41
**5→4**	**5.0 E-37**	**1.2 E-44**	**1.4 E-5**
**6→2**	**6.0 E-46**	**1.2 E-35**	0.3
**6→3**	**7.0 E-43**	**3.6 E-55**	**0.008**
**6→4**	**5.9 E-38**	**1.5 E-41**	**5.0 E-13**

Supplemental Table 15 TA-RNN F2 scores for the ablation of dual-level attention and time embedding. A-RNN: TA-RNN without time embedding nor elapsed time. T-RNN: TA-RNN without dual-attention. TA-RNN was evaluated on NACC after training using two, three, five, and six preceding visits in ADNI. Best F2 score in each case (column) is shown in bold.

Model \ Scenario	2→1	3→1	5→1	6→1
**A-RNN**	0.885 ± 0.021	0.921 ± 0.014	0.961 ± 0.008	0.968 ± 0.005
**T-RNN**	0.886 ± 0.010	0.927 ± 0.009	**0.965 ± 0.005**	0.972 ± 0.002
**TA-RNN (Proposed)**	**0.893 ± 0.014**	**0.931 ± 0.007**	0.964 ± 0.004	**0.973 ± 0.002**

Supplemental Table 16 TA-RNN-AE F2 for ablation of the dual-level attention and time embedding. A-RNN-AE: TA-RNN-AE without time embedding nor elapsed time. T-RNN-AE: TA-RNN-AE without dual-attention. TA-RNN-AE was evaluated on held-out samples in ADNI after training using two, three, five, and six preceding visits in ADNI, respectively. Best F2 score for each case (row) is shown in bold.

Scenario \ Model	A-RNN-AE	T-RNN-AE	TA-RNN-AE (Proposed)
**2→2**	0.832 ± 0.020	0.824 ± 0.033	**0.834 ± 0.022**
**2→3**	0.762 ± 0.048	**0.769 ± 0.060**	**0.769 ± 0.047**
**2→4**	0.785 ± 0.033	0.794 ± 0.054	**0.798 ± 0.038**
**3→2**	0.793 ± 0.045	0.792 ± 0.046	**0.805 ± 0.042**
**3→3**	**0.822 ± 0.029**	0.807 ± 0.045	0.812 ± 0.039
**3→4**	**0.847 ± 0.042**	0.844 ± 0.048	0.837 ± 0.054
**5→2**	0.860 ± 0.039	0.846 ± 0.043	**0.867 ± 0.042**
**5→3**	0.807 ± 0.104	**0.811 ± 0.095**	0.809 ± 0.096
**5→4**	**0.820 ± 0.109**	0.804 ± 0.117	0.819 ± 0.112
**6→2**	0.838 ± 0.091	0.836 ± 0.079	**0.842 ± 0.080**
**6→3**	0.847 ± 0.114	0.852 ± 0.080	**0.856 ± 0.094**
**6→4**	0.800 ± 0.101	0.799 ± 0.102	**0.805 ± 0.097**

Supplemental Table 17 TA-RNN-AE F2 for ablation of the dual-level attention and time embedding. A-RNN-AE: TA-RNN-AE without time embedding nor elapsed time. T-RNN-AE: TA-RNN-AE without dual-attention. TA-RNN-AE was evaluated on NACC after training using two, three, five, and six preceding visits in AND, respectively. Best F2 score for each case (row) is shown in bold.

Scenario \ Model	A-RNN-AE	T-RNN-AE	TA-RNN-AE (Proposed)
**2→2**	0.880 ± 0.023	0.895 ± 0.011	**0.908 ± 0.015**
**2→3**	0.880 ± 0.013	0.884 ± 0.009	**0.898 ± 0.010**
**2→4**	0.920 ± 0.050	0.894 ± 0.036	**0.920 ± 0.058**
**3→2**	0.914 ± 0.012	0.917 ± 0.009	**0.922 ± 0.014**
**3→3**	0.916 ± 0.030	0.910 ± 0.030	**0.942 ± 0.022**
**3→4**	0.950 ± 0.026	0.922 ± 0.032	**0.955 ± 0.014**
**5→2**	**0.962 ± 0.008**	0.958 ± 0.007	0.959 ± 0.007
**5→3**	0.955 ± 0.007	0.953 ± 0.008	**0.955 ± 0.008**
**5→4**	0.946 ± 0.009	0.947 ± 0.016	**0.949 ± 0.009**
**6→2**	0.963 ± 0.008	0.960 ± 0.009	**0.969 ± 0.005**
**6→3**	0.945 ± 0.016	0.951 ± 0.006	**0.952 ± 0.004**
**6→4**	0.956 ± 0.016	0.950 ± 0.028	**0.959 ± 0.013**

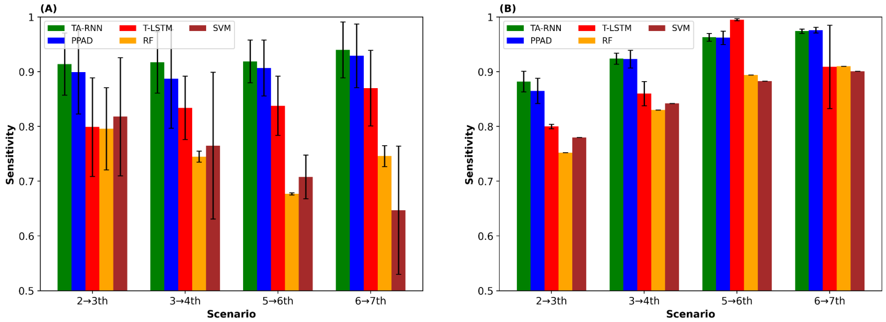

Supplemental Fig. 1 Sensitivity scores for TA-RNN models predicting conversion to AD at the next visit. (A) Models tested on held-out samples in ADNI after training using 2, 3, 5, and 6 visits in ADNI, respectively. (B) Models tested on NACC after training using 2, 3, 5, and 6 visits in ADNI, respectively.

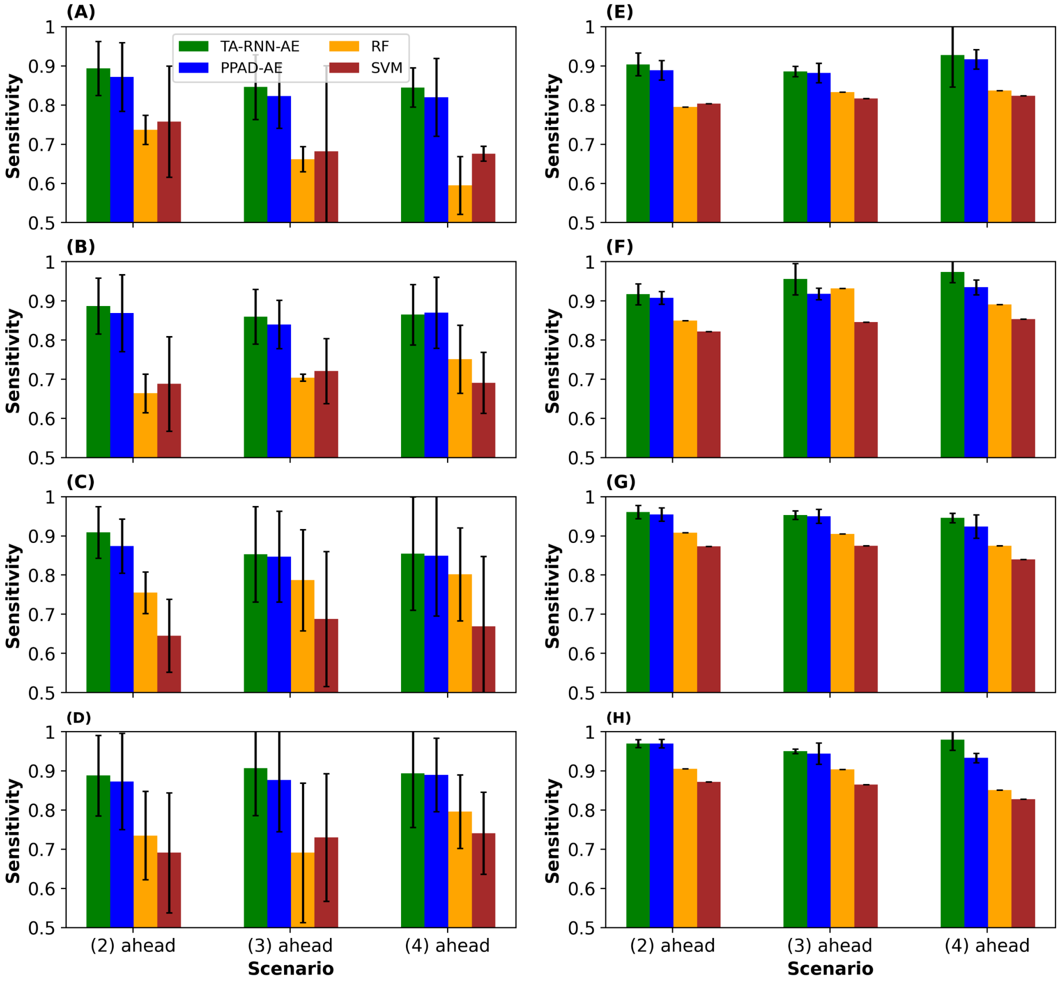

Supplemental Fig. 2 Sensitivity scores for TA-RNN-AE models predicting conversion to AD at the next second, third, and fourth visits ahead. (A, B, C, and D) Models tested on held-out samples in ADNI after training using 2, 3, 5, and 6 visits in ADNI, respectively. (E, F, G, and H) Models tested on NACC after training using 2, 3, 5, and 6 visits in ADNI, respectively.
